# Integrative analysis of DNA methylation and gene expression data for the diagnosis and underlying mechanism of Parkinson’s disease

**DOI:** 10.3389/fnagi.2022.971528

**Published:** 2022-08-18

**Authors:** Ding Li, Jiaming Liang, Wenbin Guo, Yongna Zhang, Xuan Wu, Wenzhou Zhang

**Affiliations:** ^1^Department of Pharmacy, The Affiliated Cancer Hospital of Zhengzhou University, Henan Cancer Hospital, Zhengzhou, China; ^2^Henan Engineering Research Center for Tumor Precision Medicine and Comprehensive Evaluation, Henan Cancer Hospital, Zhengzhou, China; ^3^Henan Provincial Key Laboratory of Anticancer Drug Research, Henan Cancer Hospital, Zhengzhou, China; ^4^Department of Internal Medicine, The Second Affiliated Hospital of Guangzhou Medical University, Guangzhou, China; ^5^Department of Pathology, Pingtan Comprehensive Experimental Area Hospital, Fuzhou, China; ^6^Academy of Medical Science, Zhengzhou University, Zhengzhou, China

**Keywords:** neurodegenerative disease, Parkinson’s disease, DNA methylation, methylation-driven gene, diagnostic signature

## Abstract

**Background:**

Parkinson’s disease (PD) is the second most common progressive neurodegenerative disorder and the leading cause of disability in the daily activities. In the management of PD, accurate and specific biomarkers in blood for the early diagnosis of PD are urgently needed. DNA methylation is one of the main epigenetic mechanisms and associated with the gene expression and disease initiation of PD. We aimed to construct a methylation signature for the diagnosis of PD patients, and explore the potential value of DNA methylation in therapeutic options.

**Materials and methods:**

Whole blood DNA methylation and gene expression data of PD patients as well as healthy controls were extracted from Gene Expression Omnibus database. Next, differentially expressed genes (DEGs) and differentially methylated genes (DMGs) between PD patients and healthy controls were identified. Least absolute shrinkage and selection operator cox regression analysis was carried out to construct a diagnostic signature based on the overlapped genes. And, the receiver operating characteristic (ROC) curves were drawn and the area under the curve (AUC) was used to assess the diagnostic performance of the signature in both the training and testing datasets. Finally, gene ontology and gene set enrichment analysis were subsequently carried out to explore the underlying mechanisms.

**Results:**

We obtained a total of 9,596 DMGs, 1,058 DEGs, and 237 overlapped genes in the whole blood between PD patients and healthy controls. Eight methylation-driven genes (HIST1H4L, CDC42EP3, KIT, GNLY, SLC22A1, GCM1, INO80B, and ARHGAP26) were identified to construct the gene expression signature. The AUCs in predicting PD patients were 0.84 and 0.76 in training dataset and testing dataset, respectively. Additionally, eight methylation-altered CpGs were also identified to construct the CpGs signature which showed a similarly robust diagnostic capability, with AUCs of 0.8 and 0.73 in training dataset and testing dataset, respectively.

**Conclusion:**

We conducted an integrated analysis of the gene expression and DNA methylation data, and constructed a methylation-driven genes signature and a methylation-altered CpGs signature to distinguish the patients with PD from healthy controls. Both of them had a robust prediction power and provide a new insight into personalized diagnostic and therapeutic strategies for PD.

## Introduction

As the second most diagnosed neurodegenerative disease, Parkinson’s disease (PD) is characteristic by a complex, age-related disease with more than six million patients worldwide and is the main cause of neurological dysfunction (Bloem et al., 2021). Currently, the diagnosis of PD is mainly based on clinical criteria, which have been updated many times to improve the diagnostic accuracy (Tolosa et al., 2021). The advances of gene microarray technology allow researchers to rapidly measure the expression data of thousands of genes in various diseases, helping to gain a deeper understanding of disease pathogenesis at the genetic level (Behzadi and Ranjbar, 2019). Given that specific and accurate molecular biomarkers could greatly contribute to the early diagnosis and therapy will have a greater chance of success in the early stages of disease. Thus, there is an urgent need to identify potential biomarkers for the diagnosis of PD.

Genetic variants and epigenetic changes play crucial roles in the initiation and progression of PD by through affecting endosomal, lysosomal, and mitochondrial function in pathophysiology (Dutta et al., 2021). The altered epigenetic modification or abnormal expression of PD-related genes, such as SNCA, LRRK2, MAPT, and GBA, have been reported to be closely related to PD (Bloem et al., 2021). Abnormal deposition of SNCA/α-synuclein is verified to be associated with the pathogenesis of PD (Ho et al., 2020). And all SNCA mutations were associated with the earlier age of onset and faster disease progression (Jowaed et al., 2010). Furthermore, hypomethylation of the SNCA promotor region has been reported in substantia nigra of PD patients (Matsumoto et al., 2010). The mutations in LRRK2 associated with increased kinase activity are the most common cause of autosomal dominant PD (Tolosa et al., 2020). Genome-wide association studies have implicated MAPT is a major susceptibility locus for idiopathic PD (Mata et al., 2014). Furthermore, the hypermethylation of the MAPT is neuroprotective by reducing MAPT expression (Coupland et al., 2014). Variants in GBA, encoding the enzyme glucocerebrosidase, are closely related to Lewy body diseases including PD and Lewy body dementia (Blauwendraat et al., 2020). Epigenomic changes associated with other genes including hypomethylation of NPAS2 and CYP2E1, and hypermethylation of PGC1-α, have also been implicated in PD (Lin et al., 2012).

Epigenetic mechanism, particularly DNA methylation, plays an important role in the molecular etiology of neurodegenerative diseases, including PD. DNA methylation in PD-related genes have been widely studied to explore the mechanisms in disease progression and identify potential biomarkers for early diagnosis (Kaut et al., 2012). Initial studies have explored the correlations between the regulated genes and DNA methylation in PD brain tissue (Nalls et al., 2014; Young et al., 2019). However, recent studies have described the concordant DNA methylation patterns between brain tissue and blood sample (Masliah et al., 2013; Henderson-Smith et al., 2019). While the blood sample is less invasive and easier to obtain, the blood-based biomarkers confer a number of advantages compared with the tissue-based ones (Chahine et al., 2014). And, the previous researches have revealed that some reliable biomarkers for PD also exist in blood (Su et al., 2015). Here, we conducted an integrative analysis of gene expression data and DNA methylation data based on the 5′-C-phosphate-G-3′ (CpGs) in blood between PD patients and healthy controls to identify the molecules as well as their epigenetic changes underlying PD and constructed two diagnostic signatures to distinguish the patients with PD from healthy controls.

## Materials and methods

### Data collection and procession

The DNA methylation dataset GSE145361 (1,001 PD patients and 973 health controls), and the gene expression dataset GSE99039 (205 PD patients and 233 normal blood samples) were downloaded from Gene Expression Omnibus (GEO) database^1^ on May 10, 2022. In addition, the blood DNA methylation dataset GSE111629 (335 PD patients and 237 normal blood samples), and the blood gene expression dataset of GSE6613 with 50 PD patients and 23 healthy controls were also downloaded from GEO to validate the accuracy and specificity of our signature. All the above data was downloaded using R package “GEOquery” and then preprocessed using the method described in the previous study (Henderson-Smith et al., 2019; Wang et al., 2019).

### Differential methylation and expression analysis

The differential methylation and expression analyses were performed using the method previously described (Wang et al., 2019). The gene methylation level in this study was measured according to CpGs. The differentially methylated genes (DMGs) between PD patients and normal controls in the GSE145361 dataset were identified with the thresholds of adjusted *p*-value <0.001 and fold-change >1 using R package “ChAMP.” We identified the differentially expressed genes (DEGs) between PD patients and normal controls in the GSE99039 dataset using R package “limma” with the cutoff value of adjusted *p* < 0.05 and fold-change >1.

### Construction of the Parkinson’s disease diagnostic signature based on overlapped genes

After the overlapping analysis based on the DMGs and DEGs of the PD patients compared to the normal controls, we constructed the diagnostic signature through applying least absolute shrinkage and selection operator (LASSO) Cox regression analysis and stepwise logistics regression to these overlapped genes of DMGs and DEGs to eliminate the genes highly correlated with each other to avoid overfitting. Finally, the signature was constructed with eight DNA methylation-driven genes and their coefficients.

The receiver operating characteristic (ROC) curves were drawn and area under the curve (AUC) was used to measure the performance of the gene signature in the diagnosis of PD using R package “pROC.”

### Functional enrichment analysis of differentially expressed DNA methylated genes

In order to explore the potential molecular mechanism of the differentially expressed DNA methylation-driven genes, we performed Gene ontology (GO) analysis under three terms, including biological process (BP), molecular function (MF), and cellular component (CC), using R package “clusterProfiler” (Chen et al., 2017; Wang et al., 2019; Kanehisa et al., 2021). A adjust *p* value <0.05 was set as the cutoffs of different parameters.

### Construction of the Parkinson’s disease diagnostic signature based on the DNA methylation sites

The 77 DNA methylation sites of the eight signature genes developed in GSE99039 were used as candidate sites to construct the signature. Then, in the GSE145361 dataset, LASSO analysis was carried out in the candidate sites with the R package ‘glmnet’. Finally, the signature was constructed with eight methylation sites and their coefficients. We used the AUCs of ROC curves to measure the quality of the methylation site signature in the diagnosis of PD based on the R package “pROC.”

### Validation of the gene expression and methylation site signatures in the testing datasets

To further validate the accuracy and specificity of the gene expression and methylation site signatures, we calculated the risk score of each sample based on the signature and used the AUCs of ROC curves to measure the diagnostic value in the GSE6613 and GSE111629 datasets.

### Gene set enrichment analysis

Gene set enrichment analysis (GSEA) was performed to analyze the enrichment of datasets between high- and low-expression groups of hub genes, according to the gene sets files from the KEGG databases. A adjust *P* value <0.05 was set as the cutoffs of different parameters.

### Statistical analysis

The R software version 3.6.1 and R Studio software were used to perform the statistical analyses and figures output. The false discovery rate (FDR) was used to adjust the *p*-value obtained by the Mann–Whitney *U* test. Adjusted *p*-value <0.001 were set as cutoff criteria for DMGs, and adjusted *p*-value <0.05 as threshold for DEGs. Adjust *P* value <0.05 was set as a cutoff value to identify significant biological pathways in GO and GESA analysis. Student’s *t* tests were used to determine statistical significance among two groups.

## Results

### Identification of the differentially methylated genes

**FIGURE 1:**
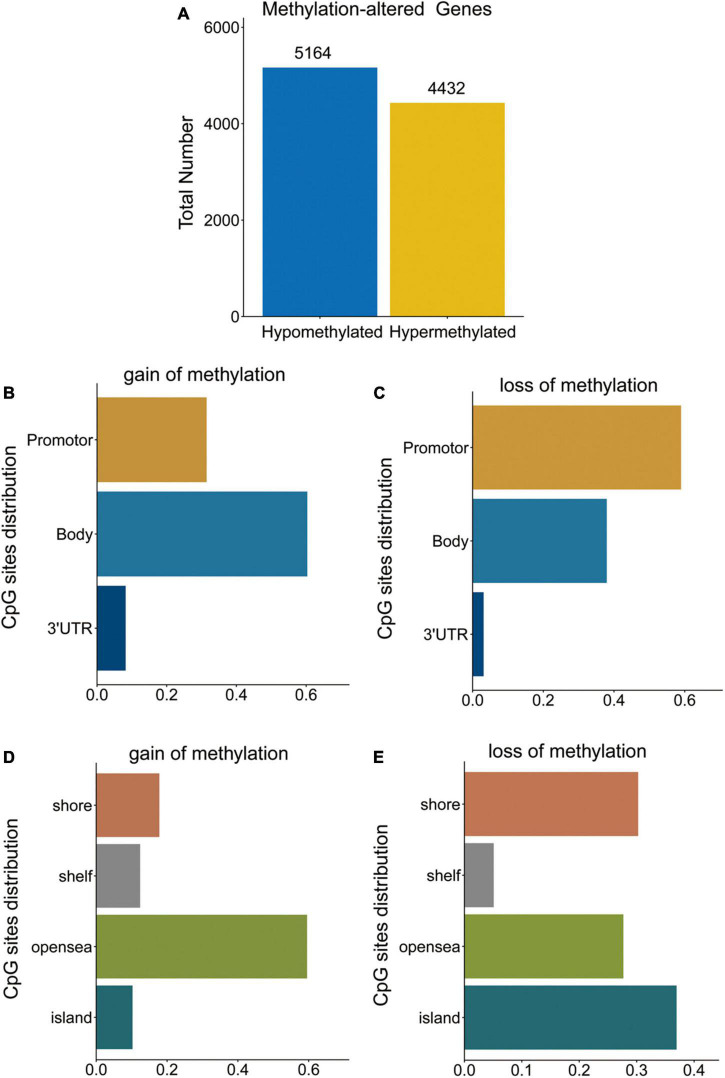
DMGs in PD patients. **(A)** Bar plot for DMGs in PD patients and healthy controls. **(B,C)** Distribution of DNA methylation changes in all genomic compartments. **(D,E)** Distribution of DNA methylation changes in varying CpG content and neighborhood context.

To identify the DMGs based on the whole blood sample between PD patients and normal controls, GSE145361 dataset with a large sample size (1,001 PD patients and 973 health controls) was downloaded from the GEO database. We obtained a total of 9,596 DMGs, in which 5,164 DMGs are hypo-methylated and 4,432 DMGs are hyper-methylated (Figure 1A).

Distribution of hypo- and hyper-methylated sites in genomic regions relative to transcription start sites (TSSs) and CpG islands are shown in Figures 1B–E, respectively. The hyper-methylated CpGs notably tended to be located in gene bodies (60.3%) and CpGs open sea (59.5%). While, hypo-methylated CpGs notably tended to be located in the promoters (TSS1500, TSS200, 5UTR, and 1stExon) (59.0%) and CpG islands (37.0%).

### Identification of the differentially expressed genes

**FIGURE 2:**
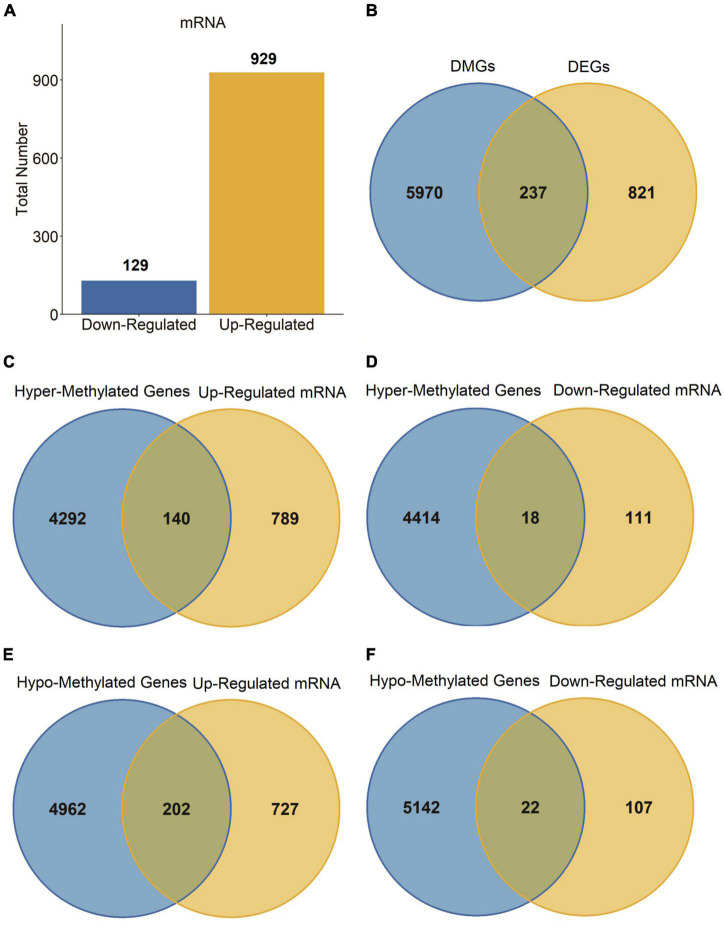
DEGs in PD patients. **(A)** Bar plot for DEGs in PD patients and healthy controls. **(B–F)** Venn diagram of DMGs and DEGs.

Next, we carried out a differential expression analysis to identify genes altered in PD patients. A linear model was applied to determine the DEGs based on the whole blood sample of 205 PD patients compared to 233 normal controls from the GSE99039 dataset. We identified 1,058 significant DEGs, including 129 downregulated genes and 929 upregulated genes (Figure 2A). Furthermore, we cross-linked DMGs and DEGs to determine 237 differentially methylation-driven genes (Figure 2B and Supplementary Table 1). To evaluate the correlation between expression levels and methylation levels of the overlapped genes, we assigned these 237 overlapped genes to four groups: hypermethylated and upregulated gene group (*n* = 140, Supplementary Table 2), hypermethylated and downregulated gene group (*n* = 18, Supplementary Table 3), hypomethylated and upregulated gene group (*n* = 202, Supplementary Table 4), and hypomethylated and downregulated gene group (*n* = 22, Supplementary Table 5). As shown in Figures 2C–F, the hypo-up genes were notably more than other group genes, which suggested that hypomethylation might be the key epigenetic modification involved in PD.

### Functional enrichment analysis of differentially expressed DNA methylation-driven genes

**FIGURE 3:**
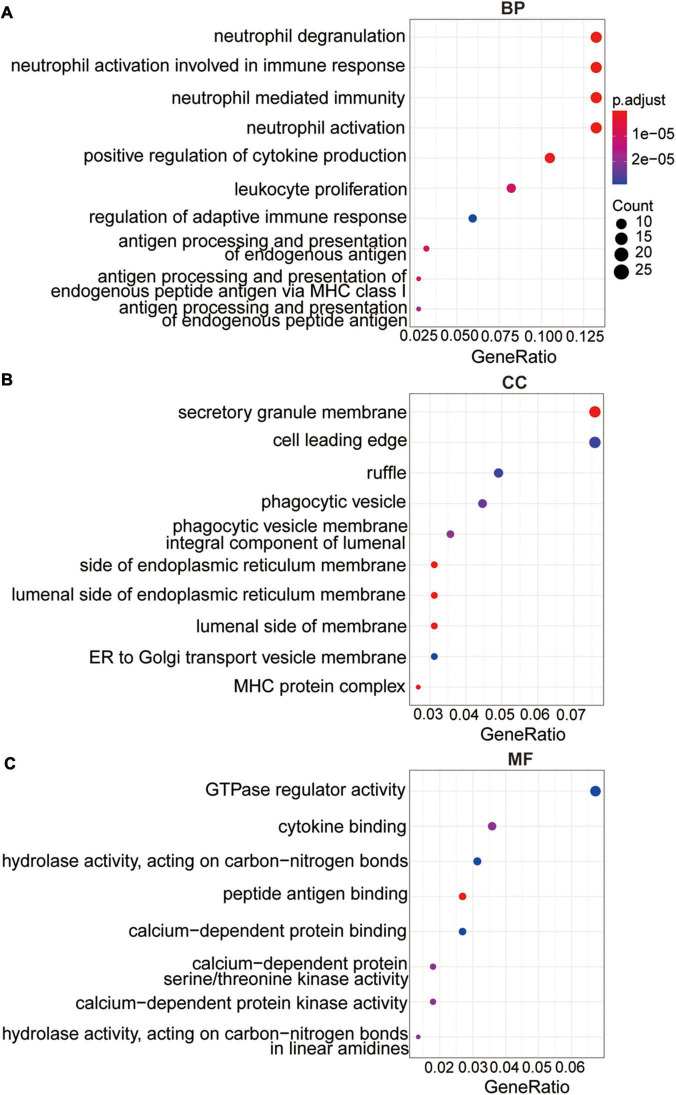
The functional analysis of the methylation-driven genes. **(A)** Top 10 of biological process enrichment. **(B)** Top 10 of cellular component enrichment. **(C)** Top 10 of molecular function enrichment.

To further explore the underlying mechanism of differentially expressed DNA methylation-driven genes, we performed GO analysis based on these 237 overlapped genes. The GO analysis revealed these overlapped genes were primarily enriched in BP terms, including neutrophil activation involved in immune response, neutrophil activation, positive regulation of cytokine production, neutrophil mediated immunity, antigen processing and presentation of endogenous antigen (Figure 3A). CC terms include integral component of lumenal side of endoplasmic reticulum membrane, secretory granule membrane, lumenal side of membrane, MHC protein complex, phagocytic vesicle, and cell leading edge (Figure 3B). MF terms include peptide antigen binding, calcium-dependent protein serine/threonine kinase activity, cytokine binding, hydrolase activity, calcium-dependent protein binding, and GTPase regulator activity (Figure 3C).

### Construction and validation of the diagnostic signature based on eight methylation-driven genes

**FIGURE 4:**
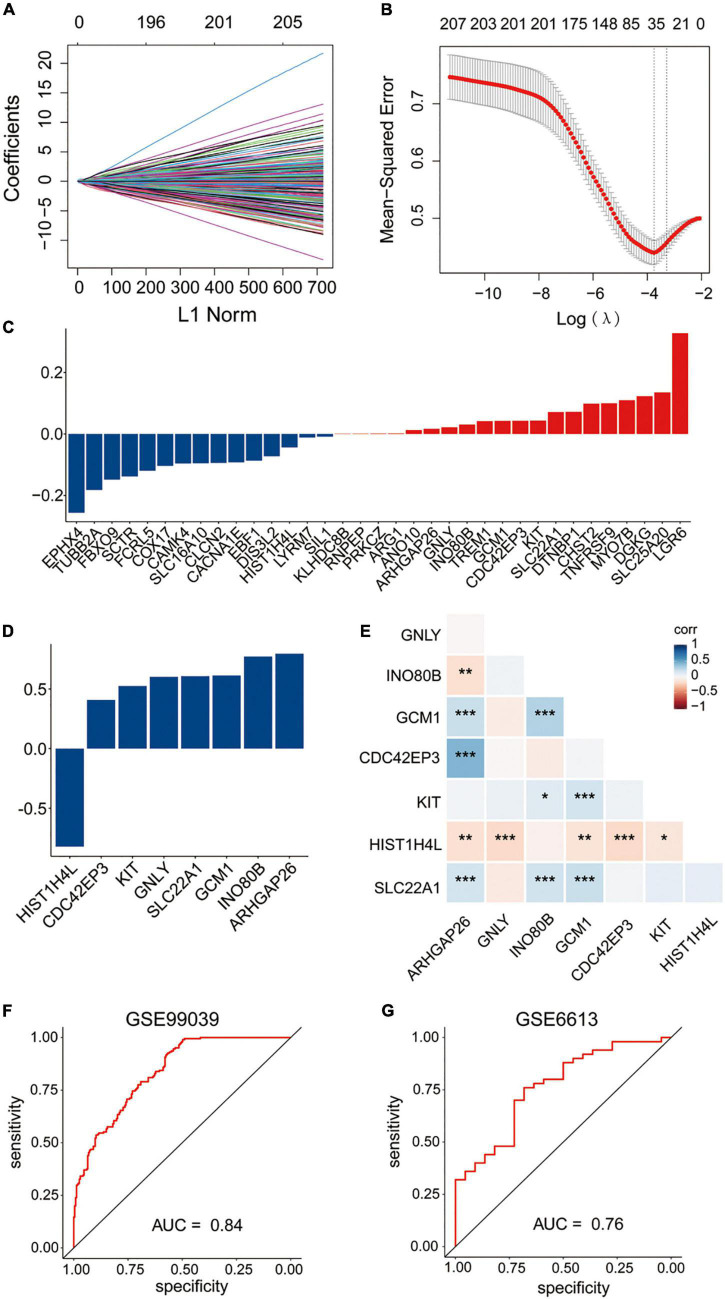
Construction of the diagnostic signature based on methylation-driven genes. **(A,B)** LASSO regression was performed to calculate the coefficients **(A)** and minimum criteria **(B)**. **(C)** Coefficients of 35 methylation-driven genes selected by LASSO regression. **(D)** Coefficients of eight methylation-driven genes in the signature selected by the stepwise logistic regression analysis. **(E)** Spearman correlation analysis of the eight genes. **(F)** ROC curve of the signature in the training set GSE99039. **(G)** ROC curve of the signature in the testing set GSE6613.

Least absolute shrinkage and selection operator regression and stepwise logistic regression analysis were applied to these 237 overlapped genes model to determine the most accurate predictive methylation-driven genes. Finally, eight methylation-driven genes HIST1H4L (H4 Clustered Histone 13), CDC42EP3 (CDC42 Effector Protein 3), KIT (receptor tyrosine kinase), GNLY (Granulysin), SLC22A1 (Solute Carrier Family 22 Member 1), GCM1 (Glial Cells Missing Transcription Factor 1), INO80B (INO80 Complex Subunit B), and ARHGAP26 (Rho GTPase Activating Protein 26) were identified. Subsequently, the signature was constructed based on the expression level and the relative coefficient of each signature gene (Figures 4A–E). The risk scoring formula was as following: risk score = (0.796331667 × ARHGAP26) + (0.772781522 × INO80B) + (0.613379779 × GCM1) + (0.608204371 × SLC22A1) + (0.602134411 × GNLY) + (0.524810413 × KIT) + (0.408771389 × CDC42EP3) + (−0.82230679 × HIST1H4L).

To evaluate the performance of the gene expression signature in the diagnosis of PD, the ROC curve was plotted and AUC achieved 0.84 when applied to the training dataset GSE99039 (Figure 4F). The AUC was 0.76 when applied to the other independent PD-associated blood gene expression dataset GSE6613 (Figure 4G), which demonstrated that the signature had accuracy predictive power for PD patients.

### Gene set enrichment analysis of the high- and low-risk groups based on the diagnostic signature

**FIGURE 5:**
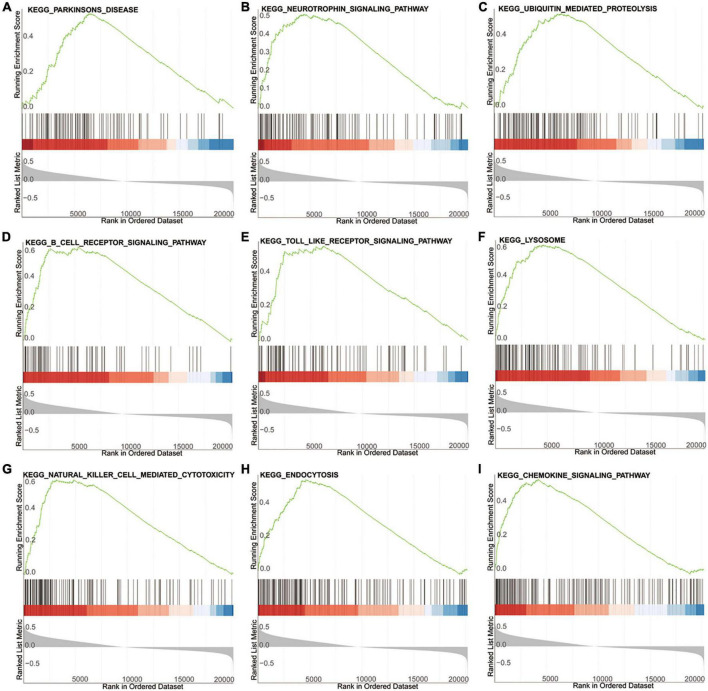
GSEA of the high- and low-risk group based on the diagnostic signature. **(A–I)** The top KEGG signaling pathways in high-risk group.

To explore the potential biological pathways and processes involved in the molecular heterogeneity, GSEA was carried out between the two risk groups in the training dataset GSE99039. As shown in Figure 5, the top KEGG (Kyoto Encyclopedia of Genes and Genomes) signaling pathways enriched in the high-risk group were associated with PD, neurotrophic signaling pathway, ubiquitin mediated proteolysis, B cell receptor signaling pathway, Toll like receptor signaling pathway, natural killer cell mediated cytotoxicity, lysosome, endocytosis, chemokine signaling pathway. These results provided new insights into pathogenesis and prevention for PD patients.

### Construction and validation of the diagnostic signature based on DNA methylation sites

**FIGURE 6:**
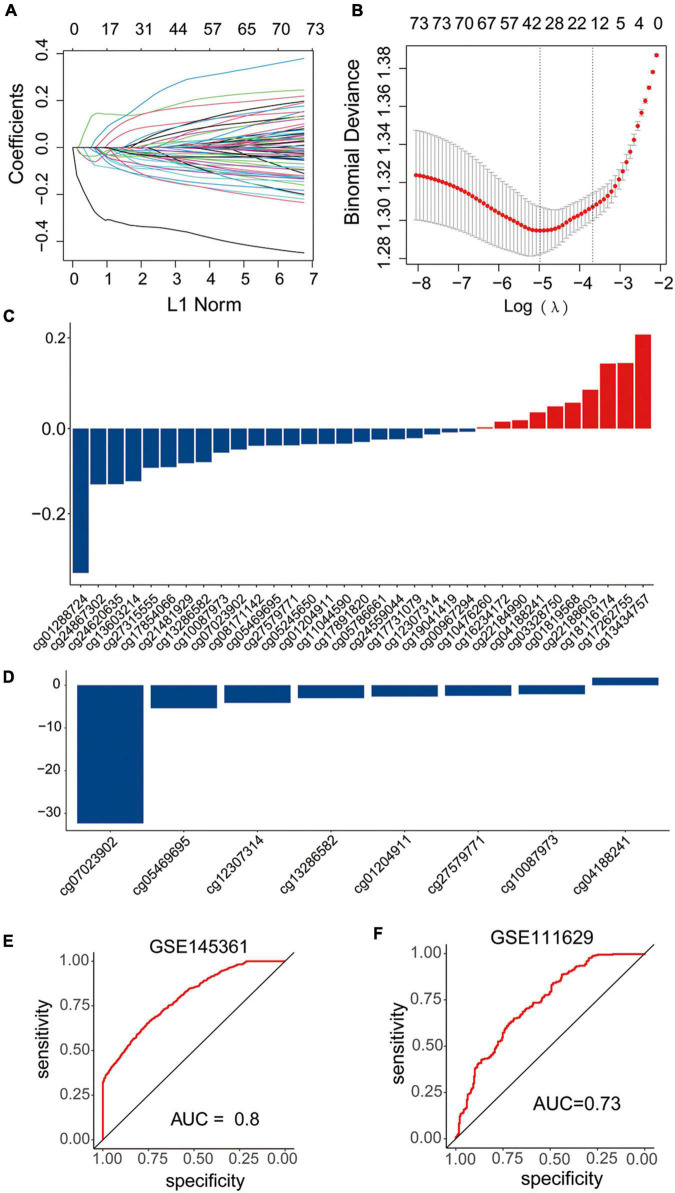
Construction and validation of the diagnostic signature based on DNA methylation sites. **(A–C)** LASSO regression was performed to calculate the **(A,B)** minimum criteria and **(C)** coefficients. **(D)** Coefficients of eight DNA methylation sites in the signature selected by the stepwise logistic regression analysis. **(E,F)** ROC curves of the signature in the training set GSE145361 and testing set GSE111629.

In the GSE145361 dataset, 77 methylation-altered CpGs associated with the eight methylation-driven genes in the gene expression signature were used in the LASSO regression model. Then, 33 CpGs obtained were further analyzed in the stepwise logistic regression model. Finally, eight CpGs were acquired to construct the signature (Figures 6A–D). The risk scoring formula was as following: risk score = (−32.323837 × cg07023902) + (−5.3875735 × cg05469695) + (4.1370905 × cg12307314) + (3.0167468 × cg13286582) + (2.6430882 × cg01204911) + (2.4635395 × cg27579771) + (2.0870986 × cg10087973) + (1.761926332 × cg04188241).

The AUC of ROC curve to distinguish the PD patients from the health controls achieved 0.8 when applied to the training dataset GSE145361 (Figure 6E). In addition, we also validated the accuracy of the CpGs signature in the other independent PD-associated blood DNA methylation dataset. The AUC of ROC curve achieved 0.73 when applied to GSE111629 dataset (Figure 6F), which indicated that the CpG signature was also efficient for PD.

## Discussion

A growing number of aberrant genome or epigenome mechanisms are associated with carcinogenesis and progression. However, the specific mechanism of methylation in neurodegenerative disease still remains poorly described. The diagnosis of PD patients mainly relies on the clinical symptoms, which hinders detection of the early stages of the disease that often had the greatest therapeutic effect. It is of great value to explore potential methylated blood biomarkers for the diagnosis of PD patients.

Omics approaches play increasingly important roles in the management of disease (Amariuta et al., 2020). Biomarkers commonly used as part of routine clinical practice can complement clinical examination and contribute to the management of various diseases. The advance of the new sequencing approach and access to some huge genetic and epigenetic databases including TCGA and GEO, are providing potential biomarkers options and the focus is shifting to a combination of several or more biomarkers, rather than a single marker that researchers have concentrated on in the past (Conway and Wong, 2020; Vincent et al., 2020; Bian et al., 2022). In this study, we conducted an integrative analysis of gene expression and DNA methylation data, and identified the DMGs and DEGs between PD patients and healthy controls. Furthermore, LASSO regression analysis was carried out to further construct the methylation-driven gene signature. Finally, an eight methylation- driven genes risk signature was developed, and the AUCs in evaluating the predictive accuracy of the signature were high (0.84 in training dataset, 0.76 in testing dataset, respectively).

Among the eight methylation-driven signature genes, ARHGAP26 is a GTPase-activating protein and inhibits the activity of Rho GTPases to affect tumorigenesis and progression of various tumors (Zhang et al., 2022). Recently, it was reported to be significantly associated with neuropsychiatric diseases and neurodegenerative diseases, including PD (Wang et al., 2022). SLC22A1 facilitates the transport, distribution, and elimination of levodopa, which is significantly associated with the occurrence of adverse events of dopaminergic treatment in PD (Redenšek et al., 2019). INO80B regulates trophoblast differentiation and embryonic stem cell self-renewal, implicating in tumorigenesis, pre-eclampsia, and avoidant personality disorder (Wang et al., 2014; Oudejans et al., 2015). GCM1 encodes a DNA-binding protein with a gcm-motif, which is associated with the epigenetic regulation of Hes5 transcription by DNA demethylation. Loss of GCM1 leads to the impaired induction of neural stem cells (Hitoshi et al., 2011). GNLY, an immune-regulator, has a close correlation with methylation and expression change, and has previously been implicated in spontaneous abortions (Novakovic et al., 2011). Oncogene KIT mediates cellular responses, such as cell survival, proliferation, and differentiation (Roskoski, 2018). It was reported that hyper-methylation in the promoter region of c-KIT proto-oncogene would result in the down-regulation of gene expression in most cancer tissues (Huang et al., 2015). CDC42EP3, one of five CDC42 effector proteins, acts as a key regulator of the activities of CDC42 (Farrugia and Calvo, 2017). Previous studies have indicated that the bio functional roles of CDC42EP3 in regulating cell shape change, actomyosin contractility and pathological fibroblast activation (Farrugia and Calvo, 2016). Additionally, CDC42EP3 is also associated with the occurrence and progression of human cancers, such as colorectal cancer (Feng et al., 2021), ovarian cancer (Yan et al., 2021), and glioma (Yang et al., 2022). HIST1H4L, known as H4 Clustered Histone 13 (H4C13), is essential nuclear proteins responsible for the nucleosome structure of the chromosomal fiber in eukaryotes. Dysregulation of HIST1H4L may lead to the alternative histone modifications and aberrant gene expression and has been identified as a senescence-related gene in lung adenocarcinoma (Wang et al., 2021; Lin et al., 2022). Overall, HIST1H4L, CDC42EP3, KIT, GNLY, GCM1, and INO80B have not been previously elucidated to be involved in PD, which provides additional insights in the underlying molecular mechanism of PD.

Despite the data came from distinct samples, and was obtained using different analytical means, some genes were overlapped between DEGs and DMGs. Notably, majority of the overlapped genes were hypomethylated and upregulated, which demonstrated that the hypomethylation was the key epigenetic modification associated with PD and hypomethylation of some PD-related genes result in the upregulation of these genes. Moreover, dominant methylation-altered regions of the genes were remarkably different. In addition, some genes had multiple dominant DNA methylation-altered regions, while others had a single dominant methylation-altered region. Therefore, to further explore the clinical value of CpGs in PD, eight significant dominant methylation-altered CpGs were used to construct a gene methylation signature. The ROC analyses demonstrated that the signature also had a superior prediction power for PD (AUCs of 0.8 in training dataset, and 0.73 in testing dataset, respectively).

To explore the underlying biological functions and signaling pathways involved in the signature, GO and GSEA analyses were performed. The results revealed that the neutrophil function and the relevant signaling pathway were significantly enriched in both GO and GSEA analyses. As the protagonists in chronic inflammation (Soehnlein et al., 2017), neutrophil activation stimulates the local and systemic inflammation, promotes proinflammatory cytokines induction and causes neuroinflammation (Kanashiro et al., 2020). Neuroinflammation has been shown to contribute to the progression of neurodegeneration in PD (Tansey and Romero-Ramos, 2019; Hirsch and Standaert, 2021). Previous evidences proved that neutrophil activation played an important role in various diseases, including cancer (Rosell et al., 2021), cardiovascular disease (Bonaventura et al., 2019), and Alzheimer’s disease (Dong et al., 2019). And many studies demonstrated that, compared with healthy controls, PD patients had a high neutrophil count, which was consistent to the results of our study (Ataç Uçar et al., 2017; Muñoz-Delgado et al., 2021). Our study showed that neutrophil activation could be an indicator of the inflammatory status and peripheral immune dysregulation in PD, but whether it is a cause or a consequence of PD progression remains unclear.

Currently, several biomarkers for PD diagnosis were available. Caldi et al. identified a miRNA signature in PD cerebrospinal fluid (Caldi Gomes et al., 2021). Shao et al. identified a metabolite panel in PD plasma samples (Shao et al., 2021). In comparison, there are also some strengthens in our study. First, we constructed the diagnostic signature based on a dataset with a large sample size. Second, we integrated the gene expression and DNA methylation data, which is more stable than a single data form. Third, the signature was based on the whole blood sample, which could be obtained with a non-invasive, convenient and easy method. Furthermore, the signature has a higher accuracy and specificity, and contains fewer genes, which is more promising for clinical application.

However, there are some limitations should be noticed in our study. First, this study was performed based on the public database and was driven by the analysis of available retrospective data. And, the optimal cutoff value was required to be defined before clinical application. In addition, our study only focused on the methylated genes. However, there are many other epigenetic modifications in disease pathology. It is of great value to integrate more modifications together. In future study, *in vivo* and/or *in vitro* experiments based on the constructed mouse model and a large number of patient blood samples are planned to validate the identified signature and elucidate the underlying mechanisms in PD.

## Conclusion

In conclusion, we performed an integrated analysis of the gene expression data and DNA methylation data, constructed a methylation-driven genes signature and a methylation-altered CpGs signature to distinguish PD patients from healthy controls. All of them have a good prediction power for PD and provide a new insight into personalized diagnostic and therapeutic strategies for PD.

## Data availability statement

The datasets presented in this study can be found in online repositories. The names of the repository/repositories and accession number(s) can be found in the article/Supplementary material.

## Author contributions

XW, DL, and WZ designed the research. JL, YZ, and WG analyzed the data. DL and XW wrote the manuscript with contributions from all the authors. All authors have read and approved the manuscript.

## Footnotes

1
http://www.ncbi.nlm.nih.gov/geo

## Abbreviations

PD: Parkinson’s disease
SNCA: Synuclein Alpha
LRRK2: Leucine Rich Repeat Kinase 2
MAPT: Microtubule Associated Protein Tau
GBA: Glucosylceramidase Beta
NPAS2: Neuronal PAS Domain Protein 2
CYP2E1: Cytochrome P450 Family 2 Subfamily E Member 1
PGC1- α: PPARG Coactivator 1 Alpha
DEGs: differentially expressed genes
DMGs: differentially methylated genes
LASSO: least absolute shrinkage and selection operator
AUC: area under the curve
GO: Gene Ontology
BP: biological process
MF: molecular function
CC: cellular component
GSEA: Gene Set Enrichment Analysis
CpGs: 5′-C-phosphate-G-3′
HIST1H4L: H4 clustered histone 13
CDC42EP3: CDC42 effector protein 3
KIT: receptor tyrosine kinase
GNLY: Granulysin
SLC22A1: solute carrier family 22 member 1
GCM1: glial cells missing transcription factor 1
INO80B: INO80 complex subunit B
ARHGAP26: Rho GTPase activating protein 26
KEGG: Kyoto Encyclopedia of Genes and Genomes.
